# Tamoxifen therapy in juvenile gigantomastia associated with PASH: a case report

**DOI:** 10.3389/fped.2026.1765982

**Published:** 2026-03-10

**Authors:** Simone Coslovich, Marina Bortul, Fabrizio Zanconati, Gianluca Tornese

**Affiliations:** 1 Department of Medicine, Surgery and Health Sciences, University of Trieste, Trieste, Italy; 2 Institute for Maternal and Child Health, IRCCS “Burlo Garofolo”, Trieste, Italy

**Keywords:** breast volume reduction, gigantomastia, non-surgical management, PASH, pediatric breast mass, tamoxifen

## Abstract

Pseudoangiomatous stromal hyperplasia (PASH) is a rare benign breast lesion in children. We report a 10-year-old girl with hormonally driven gigantomastia and recurrent mastitis, interpreted as a secondary inflammatory complication of rapid breast enlargement. Treatment with oral tamoxifen led to arrest of breast growth and resolution of inflammatory symptoms. This case supports tamoxifen as a potential non-surgical option for hormonally driven pediatric gigantomastia associated with PASH.

## Case description

A 10-year-6-month-old girl was referred for endocrinological evaluation because of recurrent mastitis in the context of gigantomastia. Thelarche had begun at approximately 10 years of age and was followed by rapid and marked breast enlargement. Approximately two months later, she developed her first episode of mastitis, which initially responded to amoxicillin–clavulanate. A further episode occurred two months later, at 10 years and 4 months of age, coinciding with menarche and showing poor response to oral antibiotic therapy.

**Figure 1:**
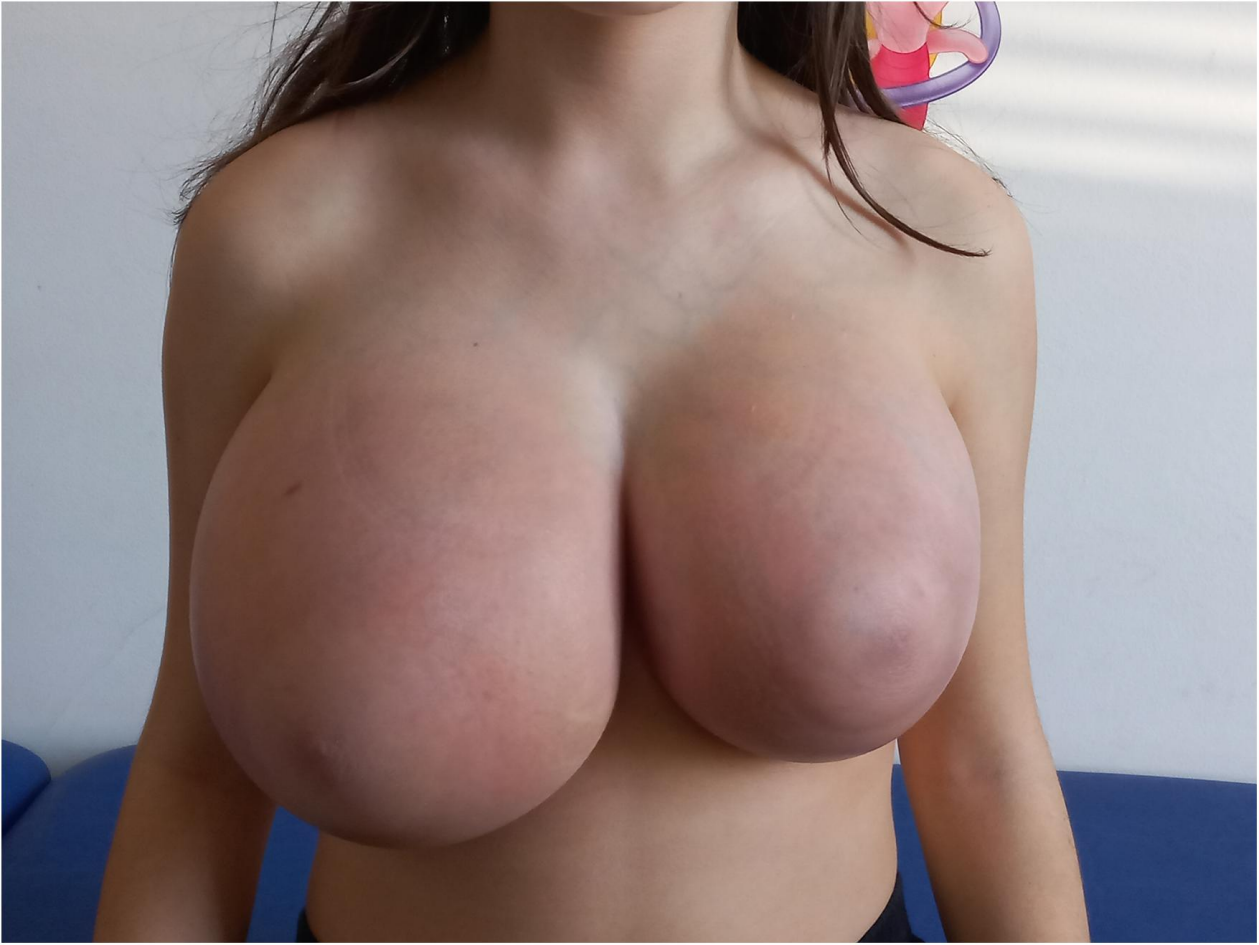
Initial presentation with marked gigantomastia, asymmetry (right breast larger), and overlying erythema.

**Figure 2:**
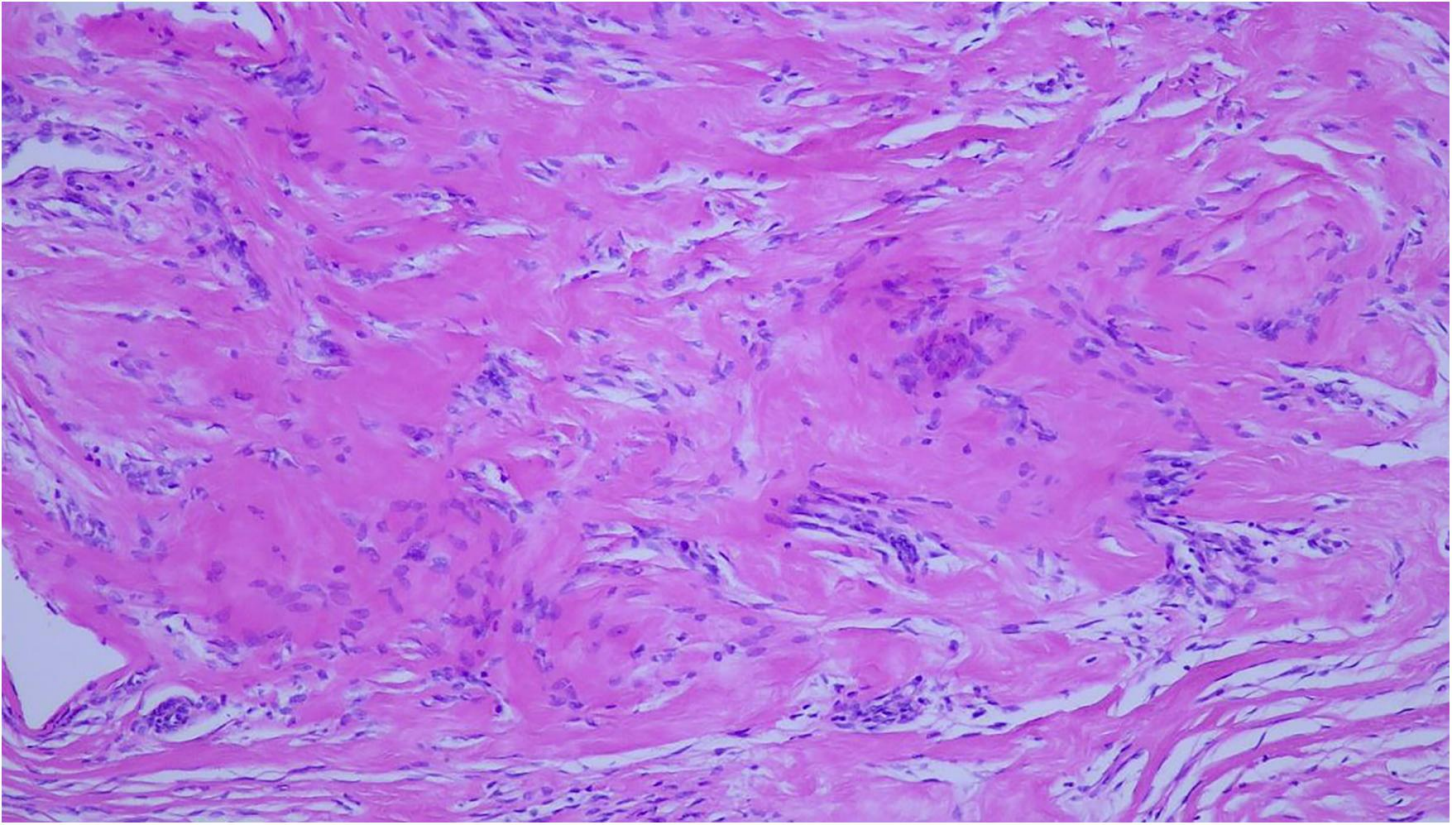
Haematoxylin and eosin–stained section showing expanded stromal areas consistent with pseudoangiomatous stromal hyperplasia (PASH).

On physical examination, breast volume was excessive for age, consistent with gigantomastia (Figure 1). The right breast was larger than the left, with overlying erythema but no discharge. Bilateral, firm, elastic nodular areas were palpable and mildly tender, without clearly defined masses. Pubertal staging corresponded to Tanner B4, PH4, A3. The remainder of the examination was unremarkable. Breast ultrasound excluded discrete masses or fluid collections, and the palpable findings were interpreted as diffuse glandular enlargement rather than true nodular lesions. Left hand/wrist x-ray (Greulich and Pyle) revealed a bone age of 12 years, about 1.5 years ahead of chronological age. Laboratory evaluation, including a full hormonal panel and tumor markers, was unremarkable except for markedly elevated estradiol levels for age (96.7 pg/mL; reference range: 20–42.8), associated with pubertal gonadotropin levels (LH: 3.8 IU/L, FSH: 3.6 IU/L), consistent with central pubertal activation. Family history was unremarkable for pubertal disorders. Both parents had normal pubertal timing; the patient's older sister had menarche at age 10. Despite the absence of discrete masses on ultrasound, core needle biopsy was performed because of rapid and asymmetric breast enlargement with recurrent inflammatory episodes, to exclude underlying stromal or neoplastic pathology. Histological examination revealed hyperplastic ducts without atypia and stromal expansion consistent with pseudoangiomatous stromal hyperplasia (PASH) (Figure 2). Immunohistochemistry demonstrated strong estrogen and progesterone receptor expression in the epithelial component, while the stroma was hormone receptor–negative. Stromal cells showed strong CD34 and vimentin positivity, variable smooth muscle actin (SMA) expression, weak or negative CD10 staining, and were negative for desmin; CD31 and D2-40 highlighted only vascular structures. Although sampling limitations inherent to core needle biopsy preclude definitive assessment of disease extent, the histological findings were consistent with a diffuse form of PASH rather than a focal lesion, in keeping with the clinical presentation of gigantomastia.

**Figure 3:**
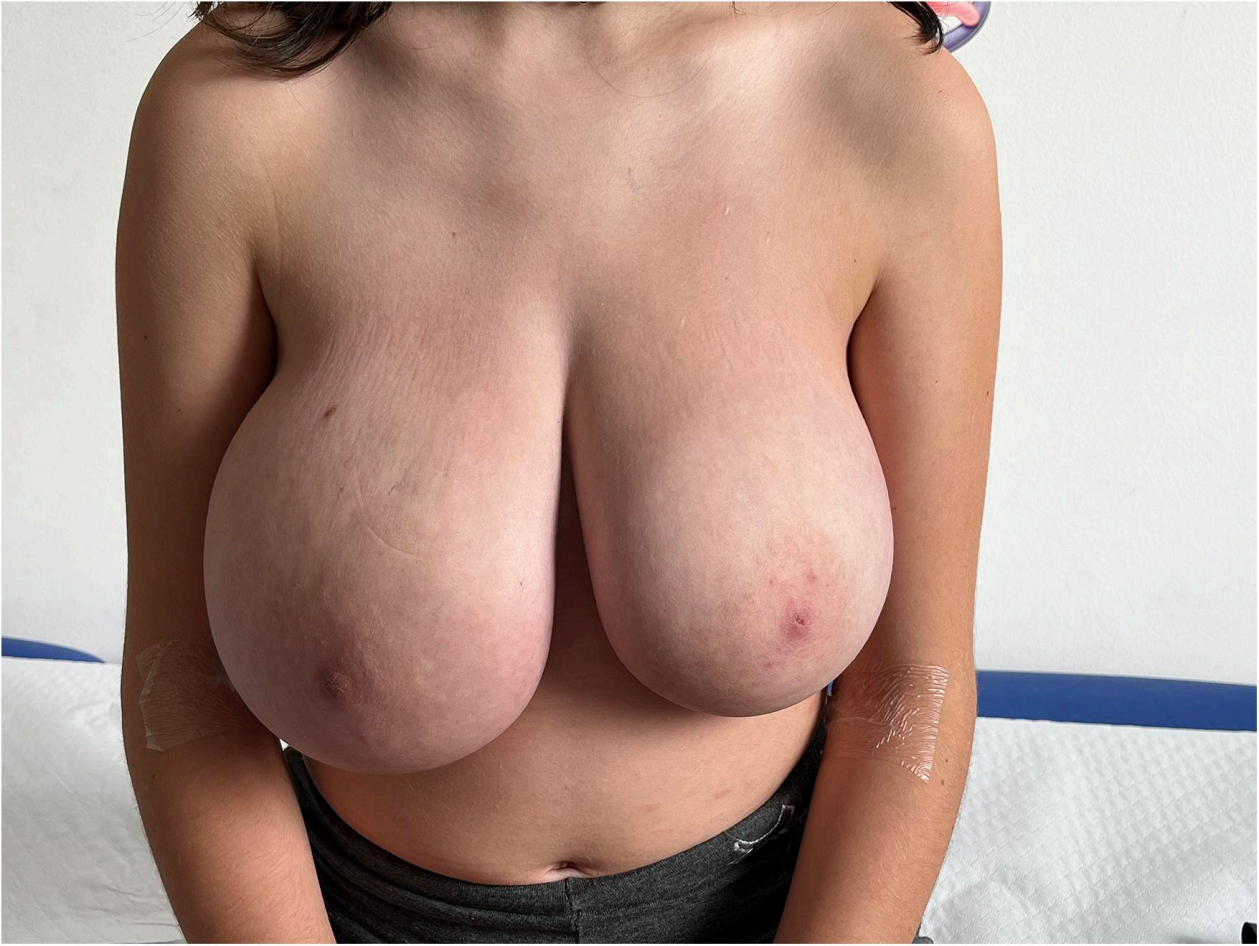
Four months after tamoxifen therapy, with significant reduction in breast swelling and tension.

Given the severity of breast enlargement, surgical reduction was discussed with the family as a potential therapeutic option. However, due to concerns regarding the patient's young age and the psychological and physical implications of early surgery, the family declined operative management at that stage. Tamoxifen was therefore initiated at 5 mg twice daily for one week and subsequently increased to 10 mg twice daily, as hormonal therapy was primarily intended to address hormonally driven gigantomastia. Pseudoangiomatous stromal hyperplasia was considered the underlying histological substrate contributing to exaggerated breast growth in the setting of pubertal hormonal activation. At 4-month follow-up, breast volume was markedly reduced, with resolution of inflammatory signs and previously tense, stretched overlying skin, along with complete resolution of breast tenderness (Figure 3). During therapy, the patient developed amenorrhea and irritability, leading her family to discontinue treatment. She was subsequently re-evaluated 6 months after therapy withdrawal: breast volume remained stable, with no signs of inflammation. In consideration of the clinical course, a reduction mammoplasty is planned in order to achieve long-term symptom control and improve quality of life.

## Discussion

Pseudoangiomatous stromal hyperplasia (PASH) is a benign breast lesion characterized by hormonally responsive stromal myofibroblast proliferation. While typically described as an incidental microscopic finding in adult women, PASH may also present as a palpable mass, mimicking fibroadenomas both clinically and radiologically. Management usually involves observation or surgical excision if symptomatic (1, 2). In rare cases, particularly in hormonally stimulated settings such as puberty, diffuse PASH can lead to rapid and excessive breast enlargement, clinically manifesting as gigantomastia. PASH is uncommon in children and adolescents, especially as a tumor-forming or diffuse lesion (3, 4).

Juvenile gigantomastia is a rare disorder characterized by disproportionate and excessive breast enlargement during puberty. Fewer than 120 cases have been reported in the literature over the past century, with a median age at presentation of approximately 18 years and an age range spanning from early adolescence to adulthood. The juvenile form accounts for nearly half of reported cases (5). Although benign, the condition may cause significant physical discomfort, recurrent inflammatory episodes, skin complications, postural problems, and considerable psychological distress. Rapid and excessive breast enlargement may predispose to secondary inflammatory manifestations, including mastitis-like episodes, even in the absence of primary bacterial infection, likely due to tissue stretching and vascular compromise (6).

In our patient, diffuse PASH likely represented the underlying pathological substrate driving hormonally mediated gigantomastia. The absence of specific radiological features on breast ultrasound, such as a well-defined mass with homogeneous echotexture and a characteristic internal vascular pattern, underscores the diagnostic challenge of PASH in children, where diffuse glandular enlargement may mimic other causes of juvenile breast hypertrophy (7, 8). The hormonal responsiveness of PASH is supported by frequent progesterone receptor expression in stromal cells and by reported clinical responses to anti-estrogen or anti-progestin therapies (9, 10). In our patient, however, receptors were positive in the epithelial component but negative in the stroma, suggesting heterogeneity in receptor distribution or sampling limitations. Early pubertal status and elevated estradiol levels further support a hormonally driven process. Although tamoxifen has been reported in the treatment of PASH, evidence supporting its efficacy in this specific condition is limited, with one published case report describing clinical improvement in an adult patient (11). In contrast, anti-estrogen therapy has been more extensively described in cases of juvenile gigantomastia, particularly to control rapid breast enlargement or prevent postoperative recurrence (12–15). Given the severity of breast hypertrophy, reduction mammoplasty was proposed as the standard therapeutic option (16). However, after multidisciplinary discussion and shared decision-making with the family, surgical intervention was deferred due to concerns regarding the patient's young age, potential psychological impact, risk of recurrence during ongoing pubertal stimulation, and possible need for repeat procedures. Medical therapy with tamoxifen was therefore initiated with the primary aim of controlling hormonally driven gigantomastia, while PASH was considered the histological contributor to this exaggerated growth pattern. In our patient, tamoxifen induced rapid clinical improvement, supporting its potential role in carefully selected pediatric cases of hormonally driven gigantomastia. Nonetheless, endocrine side effects such as amenorrhea and mood changes necessitated treatment discontinuation, highlighting the importance of individualized risk–benefit evaluation and careful monitoring in children. Although benign and not typically associated with malignancy, PASH carries a risk of recurrence, particularly when diffuse or incompletely excised (12, 17, 18). In massive cases, surgical reduction may relieve symptoms but often does not eradicate the disease, requiring long-term follow-up.

## Conclusion

Juvenile gigantomastia is a rare but potentially debilitating condition that may occasionally be associated with diffuse PASH as an underlying histological substrate. This case demonstrates the importance of a multidisciplinary approach, balancing surgical and endocrine options, while addressing the physical and psychological impact of breast pathology in growing patients. Anti-estrogen therapy may represent a temporary or adjunctive strategy in selected cases, although evidence remains limited and further studies are needed to better define optimal management in pediatric patients.

## Data Availability

The original contributions presented in the study are included in the article/Supplementary Material, further inquiries can be directed to the corresponding authors.
